# Supplementary material for: Electrochemical Evaluation of a Multi-Site Clinical Depth Recording Electrode for Monitoring Cerebral Tissue Oxygen
Source: Micromachines (Basel). 2020 Jun 28;11(7):632. doi: 10.3390/mi11070632 (PMC7407998; doi:10.3390/mi11070632)
Supplement: Supplementary file 1 [file micromachines-11-00632-s001.docx]

Supplementary Materials: Electrochemical Evaluation of a Clinical Depth Multi-Site Recording Electrode for Monitoring Cerebral Tissue Oxygen

Ana Ledo, Eliana Fernandes, Jorge E. Quintero, Greg A. Gerhardt and Rui M. Barbosa


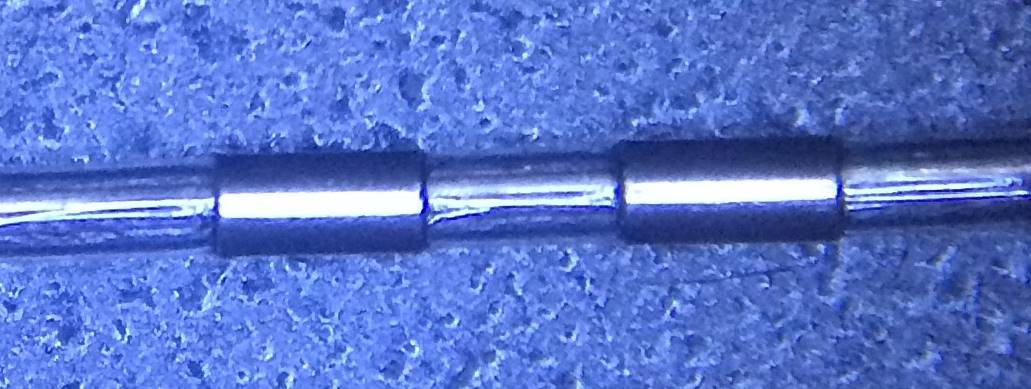

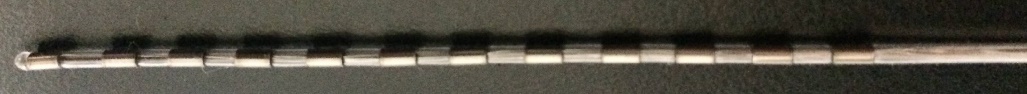

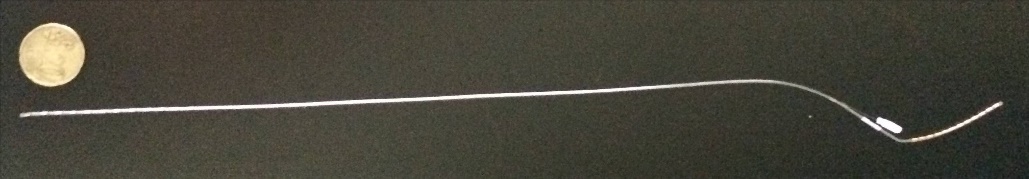


**2.5 mm**

**B**

**A**

**Figure S1.** (**A**) Photograph of the clinical depth multi-site recording electrode with 20 eurocent coin Figure 12. in line cylindrical Pt recording sites. (**B**) Detail of 2 successive Pt recording sites.

**A**


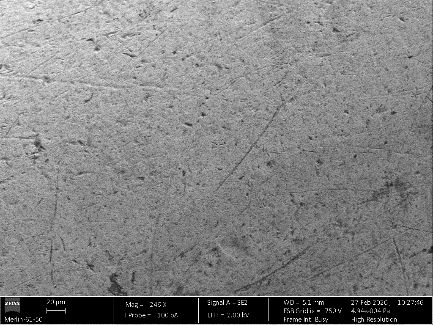

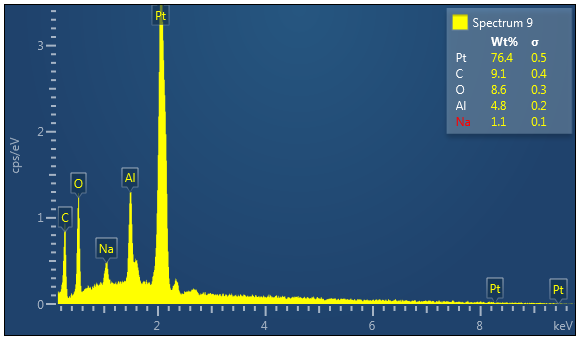

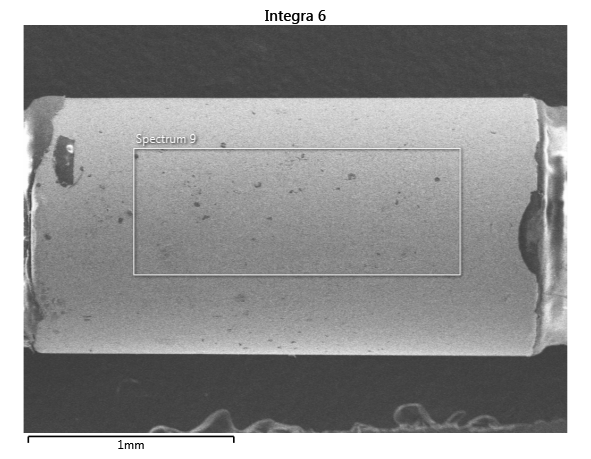

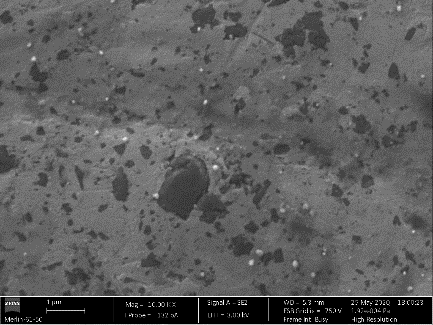

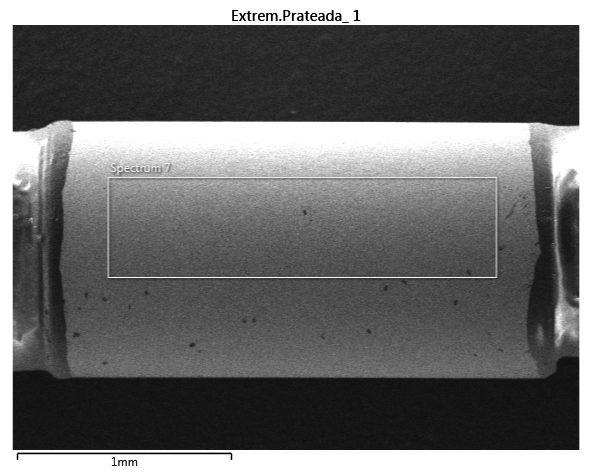

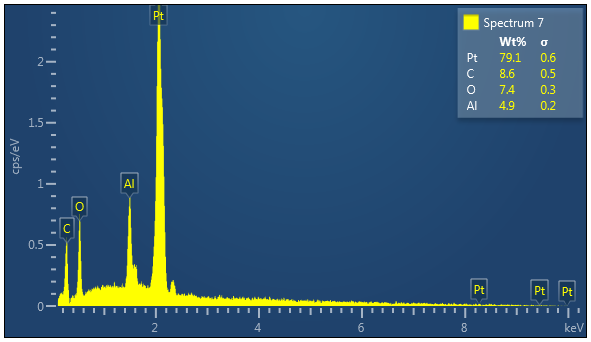

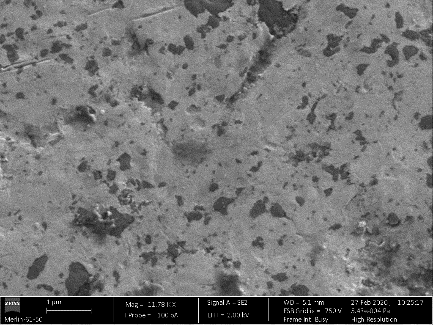

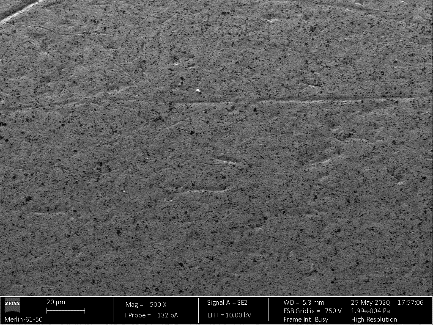


**A1**

**A2**

**A3**

**A4**

**B1**

**B2**

**B3**

**B4**

**Figure S2.** SEM micrographs and EDS elemental analysis of the surface of a new depth electrode recording site (**A**1–4) and after electrochemical evaluations (**B**1–4). Aside from the appearance of Na+ in the later (**B**4), no significant changes in surface morphology or elemental composition are observed due to *in vitro* “wear and tear”.
